# Predictive Factors for the Need of Tracheostomy in Patients With Large Vessel Occlusion Stroke Being Treated With Mechanical Thrombectomy

**DOI:** 10.3389/fneur.2021.728624

**Published:** 2021-11-26

**Authors:** Ilko L. Maier, Katarina Schramm, Mathias Bähr, Daniel Behme, Marios-Nikos Psychogios, Jan Liman

**Affiliations:** ^1^Department of Neurology, University Medical Center Göttingen, Göttingen, Germany; ^2^Department of Neuroradiology, University Hospital Magdeburg, Magdeburg, Germany; ^3^Department of Neuroradiology, Universitätsspital Basel, Basel, Switzerland

**Keywords:** ischemic stroke, mechanical thrombectomy (MT), large vessel occlusion (LVO), intensive care medicine (ICM), tracheostomy (TS)

## Abstract

**Background:** Patients with large vessel occlusion stroke (LVOS) eligible for mechanical thrombectomy (MT) are at risk for stroke- and non-stroke-related complications resulting in the need for tracheostomy (TS). Risk factors for TS have not yet been systematically investigated in this subgroup of stroke patients.

**Methods:** Prospectively derived data from patients with LVOS and MT being treated in a large, academic neurological ICU (neuro-ICU) between 2014 and 2019 were analyzed in this single-center study. Predictive value of peri- and post-interventional factors, stroke imaging, and pre-stroke medical history were investigated for their potential to predict tracheostomy during ICU stay using logistic regression models.

**Results:** From 635 LVOS-patients treated with MT, 40 (6.3%) underwent tracheostomy during their neuro-ICU stay. Patients receiving tracheostomy were younger [71 (62–75) vs. 77 (66–83), *p* < 0.001], had a higher National Institute of Health Stroke Scale (NIHSS) at baseline [18 (15–20) vs. 15 (10–19), *p* = 0.009] as well as higher rates of hospital acquired pneumonia (HAP) [39 (97.5%) vs. 224 (37.6%), *p* < 0.001], failed extubation [15 (37.5%) vs. 19 (3.2%), *p* < 0.001], sepsis [11 (27.5%) vs. 16 (2.7%), *p* < 0.001], symptomatic intracerebral hemorrhage [5 (12.5%) vs. 22 (3.9%), *p* = 0.026] and decompressive hemicraniectomy (DH) [19 (51.4%) vs. 21 (3.8%), *p* < 0.001]. In multivariate logistic regression analysis, HAP (OR 21.26 (CI 2.76–163.56), *p* = 0.003], Sepsis [OR 5.39 (1.71–16.91), *p* = 0.004], failed extubation [OR 8.41 (3.09–22.93), *p* < 0.001] and DH [OR 9.94 (3.92–25.21), *p* < 0.001] remained as strongest predictors for TS. Patients with longer periods from admission to TS had longer ICU length of stay (*r* = 0.384, *p* = 0.03). There was no association between the time from admission to TS and clinical outcome (NIHSS at discharge: *r* = 0.125, *p* = 0.461; mRS at 90 days: *r* = −0.179, *p* = 0.403).

**Conclusions:** Patients with LVOS undergoing MT are at high risk to require TS if extubation after the intervention fails, DH is needed, and severe infectious complications occur in the acute phase after ischemic stroke. These factors are likely to be useful for the indication and timing of TS to reduce overall sedation and shorten ICU length of stay.

## Introduction

Mechanical thrombectomy has been shown to be highly effective and is the standard of care for large vessel occlusion stroke (LVOS) (1). A large meta-analysis of randomized controlled trials, however, showed that >20% of patients with LVOS and mechanical thrombectomy (MT) had unfavorable outcomes with modified Rankin scores (mRS) of 4 or 5, likely requiring pronged in-patient stay with treatment on intensive care units (ICU) and increased risks for stroke and non-stroke related complications (1). Taking all ischemic stroke patients into account, it has been estimated that around 24% of this population needs ICU treatment (2).

Various complications in LVOS-patients are associated with ICU treatment and lead to unfavorable functional outcomes (3). These complications include neurological causes of decreased consciousness by large infarctions, cerebral edema, or symptomatic intracerebral hemorrhage (sICH), which also can lead to respiratory complications like pneumonia caused by stroke-associated dysphagia and again can result in hemodynamic instabilities caused by systemic inflammatory responses (4). In these scenarios, multiple factors influence the decision if-, and at which time point to perform a tracheostomy (TS) to achieve long term ventilatory support, drastically decrease sedatives and shorten prolonged orotracheal intubation, both in combination associated with increased rates of pneumonia and length of ICU stay (5). Failed extubation rates of around 17% in mixed neurological ICU (neuro-ICU) populations with acute brain injury have been reported, not only describing an association with quantitative and qualitative measurements of consciousness (e.g., a Glascow Coma Scale (GCS) of ≤ 8 points and the inability to obey commands), but also identifying multiple other factors like chronic obstructive pulmonary disease or congestive heart failure being associated with increased risk of reintubation, reflecting that conventional extubating criteria are not applicable in this patient group (6, 7). Therefore, the decision and timepoint of TS in major stroke patients should most likely be guided by clinical and functional status, emerging complications, and past medical history rather than only focusing on the state of consciousness and ventilatory function (5). If, in this context, an early TS translates into better clinical outcomes in major stroke patients is being investigated in a still unpublished randomized control trial (8), after Bösel et al. (9) showed feasibility and safety of early tracheostomy in major stroke patients in a pilot study.

Patients with LVOS, in contrast to a general population of major stroke patients, represent a well-characterized subgroup both at risk for intravenous thrombolysis (IVT)-/MT associated- as well as cardio-respiratory associated complications increasing the risk for ICU-treatment, as most patients receive general anesthesia with orotracheal intubation prior MT. In this subgroup of major stroke patients, a combination of factors increasing the risk for TS, to date, has not been systematically investigated. The aim of this study was to describe the proportion and characteristics of LVOS-patients receiving a TS after MT and to determine factors predicting the need for TS during their ICU stay.

## Materials and Methods

### Patient Population and Clinical Characteristics

We used a prospectively derived, single-center database including all patients receiving MT for LVOS treatment between 2014 and 2019 in a large, academic stroke center and being treated on a neuro-ICU. This database includes information on time metrics, imaging, intervention, patient history, and stroke- as well as non-stroke associated complications after MT. Information on the timepoint of tracheostomy, blood gases, failed extubation and pulmonary diseases were obtained retrospectively through a chart review [IntelliSpace Critical Care and Anesthesia (ICCA) information system (Koninklijke Philips N.V., 2004–2017)]. Ethics approval was sought from the Ethics Committee of the University Medicine Göttingen (13/7/15) and all patients or next of kin gave informed written consent for the anonymized use of disease-related data on hospitalization. Patients were included in the analysis if a predefined dataset on pre-stroke history (comorbidities), periinterventional data (imaging, time metrics, scores), and clinical data on the post-stroke course (e.g., infectious complications) was complete and available, which was the case in 635 (88.7%) from 716 patients included in the databank in the mentioned time period.

In the case of complete datasets, we included all patients with LVOS receiving MT with or without prior IVT. As the success of the reperfusion therapy is a major predictor for clinical outcome, we only included patients with no- or minimal perfusion (complete or near-complete vessel occlusion) of the occluded vessel on the first angiogram prior to MT [defined as modified Thrombolysis in cerebral infarction (mTICI) ≤ 1]. Therefore, patients with spontaneous reperfusion or reperfusion through IVT were excluded. All patients were treated on the Neuro-ICU of the University Medical Center Göttingen, Germany. We included all types of LVO being treated at the discretion of the neurointerventionalist performing the MT. The indication for IVT was according to the current German guidelines and all patients were treated from stroke experienced intensive care trained neurologists only. The decision for the need for a TS and its timepoint was made by ICU-trained senior consultant neurologists without the use of standard criteria or SOPs. All TSs were performed as surgical TSs by experienced Ears, Nose, and Throat (ENT) specialists. The indication for a decompressive hemicraniectomy was made in consensus with stroke experienced neurosurgeons.

### Stroke and Non-stroke Associated Complications After MT

Following complications after MT were documented and investigated: failed extubation was defined as ≥ 1 complete removal of the orotracheal tube with the need of reintubation within a 48 h period. Hospital-acquired pneumonia (HAP) was diagnosed if an infiltrate/suspicion of an infiltrate was visualized on chest X-ray in presence of at least two clinical signs such as pyogenic secretion, fever (>38°C), leukocytosis, or leukopenia (>10,000 or <4,000/l) or the detection of a pneumonia-typical pathogen in the bronchial secretion or blood (10). For the diagnosis of sepsis, the sequential (sepsis-related) organ failure assessment (SOFA) score has been used and was ≥2 in combination with positive blood cultures (11).

### Statistical Analysis

Statistical analysis was performed using SPSS 21 (IBM SPSS Statistics, Armonk, NY, USA). Characteristics of all patients are shown as *M* ± *SD* if normally distributed, and as median with interquartile range (IQR), if not. Category variables were given as absolute frequencies and percentages and examined by the Pearson Chi-Square test for statistically significant differences between the compared groups. The different groups were examined for significant differences by using independent samples *T*-test or Mann–Whitney U test, as appropriate. Uni- and multi-variate logistic regression analysis were performed for clinical and imaging factors as well as complications being unequally distributed between the TS and non-tracheostomy (nTS) group with *p* < 0.1 in a univariate pre-test. In multivariate logistic regression, the pre-identified factors for TS were included and the backward selection (Wald) was used. The clinical scores National Institute of Health Stroke Scale at discharge, mRS at discharge, and after 90 days were not included in the models and were only used to investigate an association between time from admission to TS and clinical outcome. In a final step, the model's regression coefficients of the identified predictors for TS were used to create a score, which again was analyzed using the Area Under the Receiver Operating Characteristic (AUROC) method. The cut-off score was defined as a score with maximal Youden-Index (Youdens J = sensitivity + specificity – 1). Pearson correlations have been used to investigate the strength and direction of associations between treatment times and functional outcome scores. In all procedures, a *p* < 0.05 was considered statistically significant.

## Results

From the 635 LVOS-patients included in this analysis, 40 (6.7%) required TS during their neuro-ICU stay after MT. Baseline characteristics are given in Table 1. Patients receiving TS were significantly younger [71 (62–75) vs. 77 (66–83), *p* < 0.001], had a higher NIHSS [18 (15–20) vs. 15 (10–19), *p* = 0.009], and a lower Alberta stroke program early CT score (ASPECTS) at baseline. Symptom-to-groin times were significantly longer in TS patients [268 (202–360) vs. 211 (154–284) min, *p* = 0.005], while rates of successful recanalization (mTICI≥2b 72.5% vs. 79.8%, *p* = 0.311) and final mTICI scores (*p* = 0.703) did not differ between groups. Functional outcome was significantly worse in patients with TS compared with nTS [NIHSS discharge: 19 (14–23) vs. 5 (2–11), *p* < 0.001; mRS discharge: 5 (5) vs. 3 (1–5), *p* < 0.001] and patients with TS had a longer neuro-ICU length of stay [22 (14.25–29) vs. 9 (5–14), *p* < 0.001]. Mortality did not differ between groups (22.5 vs. 28.4%, *p* = 0.470). Significantly more patients in the nTS group received IVT (22.5 vs. 37.5%, *p* = 0.019); all other ischemic stroke treatment characteristics were comparable between the groups. With the exception of a history of pulmonary embolism, which was higher in the TS group (15 vs. 3.5%, *p* = 0.005), there was no difference in rates of pulmonary comorbidities between groups.

**Table 1:** Baseline characteristics of patients with- and without tracheostomy after mechanical thrombectomy (*n* = 635).

	**Tracheostomy group (*n* = 40)**	**No tracheostomy group (*n* = 595)**	***p*-value**
**Patient characteristics and past medical history**			
Age (median, IQR)	71 (62–75)	77 (66–83)	<0.001
Sex (*n*, % male)	19 (47.5)	268 (45.0)	0.870
Arterial hypertension (*n*, %)	30 (78.9)	462 (80.8)	0.832
Hyperlipoproteinemia (*n*, %)	15 (40.5)	282 (49.7)	0.311
Diabetes Mellitus (*n*, %)	16 (43.2)	160 (28.2)	0.061
Atrial fibrillation (*n*, %)	20 (54.1)	257 (45.5)	0.395
Peripheral artery disease (*n*, %)	0 (0)	36 (6.4)	0.157
Obesity (*n*, %)	14 (38.9)	150 (26.8)	0.125
Smoking (*n*, %)	3 (8.3)	98 (17.5)	0.249
Coronary artery disease (*n*, %)	8 (21.6)	134 (23.8)	0.844
Chronic renal failure (*n*, %)	8 (22.9)	134 (23.9)	1.000
Congestive heart failure (*n*, %)	12 (30)	148 (25.3)	0.574
Pulmonary disease^*^ (*n*, %)	15 (37.5)	134 (22.5)	0.106
COPD (*n*, %)	5 (12.5)	55 (9.2)	0.414
Bronchial asthma (*n*, %)	1 (2.5)	11 (1.8)	0.545
Lung cancer (*n*, %)	0 (0)	14 (2.4)	1.000
Pulmonary emphysema (*n*, %)	0 (0)	8 (1.3)	1.000
Pulmonary embolism (*n*, %)	6 (15)	21 (3.5)	0.005
Community acquired pneumonia (*n*, %)	1 (2.5)	7 (1.2)	0.408
Pulmonary fibrosis (*n*, %)	0 (0)	3 (0.5)	1.000
Pulmonary hypertension (*n*, %)	2 (5)	15 (2.5)	0.291
**Clinical scores and imaging characteristics**			
NIHSS baseline (median, IQR)	18 (15–20)	15 (10–19)	0.009
NIHSS discharge (median, IQR)	19 (14–23)	5 (2–11)	<0.001
mRS discharge (median, IQR)	5 (5)	3 (1–5)	<0.001
mRS 90 days (median, IQR)	5 (4–6)	4 (1–6)	0.062
Favorable functional outcome (mRS ≤ 3 at 90 days) (*n*, %)	5 (12.5)	286 (48.1)	<0.001
Mortality (mRS 6 at discharge) (*n*, %)	9 (22.5)	169 (28.4)	0.470
cCT ASPECTS at baseline (median, IQR)	7 (5–8)	8 (7–9)	<0.001
cCT ASPECTS 24-h follow-up (median, IQR)	4 (2–6)	7 (5–8)	<0.001
Symptom onset to recanalization time (median min, IQR)	268 (202–360)	211 (154–284)	0.005
Successful recanalization (*n*, %)	29 (72.5)	459 (79.8)	0.311
Final mTICI score			0.703
mTICI 0	3 (7.5)	49 (8.2)	
mTICI 1	3 (7.5)	21 (3.5)	
mTICI 2a	5 (12.5)	46 (7.7)	
mTICI 2b	13 (32.5)	201 (33.8)	
mTICI 2c	6 (15)	117 (19.7)	
mTICI 3	10 (25)	140 (23.5)	
Oxygenation index (median, IQR)	345 (195–472)	376 (292–508)	0.108
**Ischemic stroke treatment characteristics**			
Side of occluded vessel (*n*, % right)	18 (50)	243 (46.6)	0.960
Site of occluded vessel			0.232
M1 (*n*, %)	20 (50)	279 (48.2)	
M2 (*n*, %)	0 (0)	85 (14.7)	
ICA proximal (*n*, %)	2 (5)	19 (3.3)	
Intracranial carotid	14 (35)	117 (20.2)	
bifurcation (*n*, %)			
BA (*n*, %)	4 (10)	56 (9.7)	
Other (*n*, %)	0 (0)	23 (4)	
Missing (*n*, %)	0 (0)	16 (2.7)	
Stroke etiology (TOAST criteria)			0.752
Large vessel	2 (5)	65 (10.9)	
atherosclerosis			
Cardioembolism	21 (52.5)	272 (45.7)	
Stroke of other	2 (5)	20 (3.4)	
determined etiology			
Stroke of undetermined	10 (25)	196 (32.9)	
etiology			
Missing (*n*, %)	5 (12.5)	42 (7.1)	
Wake up stroke (*n*, %)	5 (12.5)	63 (10.6)	0.907
Intravenous thrombolysis (*n*, %)	9 (22.5)	223 (37.5)	0.019
Type of anesthesia for mechanical thrombectomy			0.070
General anesthesia (*n*, %)	29 (72.5)	277 (46.5)	
Conscious sedation (*n*, %)	4 (10)	126 (21.2)	
Switch from conscious sedation to general anesthesia (*n*, %)	4 (10)	46 (7.7)	
Length of in-hospital stay (median days, IQR)	22 (14.25–29)	9 (5–14)	<0.001
Length of mechanical ventilation (tube, median days, IQR)	14.22 (12.42–17.36)	0.04 (0–0.46)	<0.001
Length of mechanical ventilation (total, median days, IQR)	19.92 (16.38–24.94)	0.04 (0–0.46)	<0.001
Time from admission to tracheostomy (median days, IQR)	15.54 (12.95-18.85)	n.a.	n.a.
**Postinterventional complications**			
Failed extubation (*n*, %)	15 (37.5)	19 (3.2)	<0.001
Hospital acquired pneumonia (*n*, %)	39 (97.5)	224 (37.6)	<0.001
Sepsis (*n*, %)	11 (27.5)	16 (2.7)	<0.001
Any ICH (*n*, %)	15 (37.5)	83 (13.9)	<0.001
Symptomatic ICH^#^ (*n*, %)	5 (12.5)	22 (3.9)	0.026
Subarachnoidal hemorrhage (*n*, %)	11 (27.5)	64 (10.8)	0.003
Decompressive hemicraniectomy (*n*, %)	19 (51.4)	21 (3.8)	<0.001

*COPD, chronic obstructive pulmonary disease; NIHSS, National institute of health stroke scale; mRS, modified Rankin scale; ASPECTS, Alberta stroke program early CT score; mTICI, modified Thrombolysis in cerebral infarction scale; M1/2, medial cerebral artery in its M1 or M2 segment; ICA, internal carotid artery; BA, basilar artery, TOAST, Trial of Org 10172 in Acute Stroke Treatment; ICH, intracerebral hemorrhage*.

* *Pulmonary disease includes COPD, bronchial asthma, lung cancer, pulmonary emphysema, pulmonary fibrosis, pulmonary hypertension, and pulmonary embolism; Successful recanalization was defined as mTICI ≥ 2b*.

# *Symptomatic intracerebral hemorrhage was defined as any intraparenchymal hemorrhage leading to a clinical deterioration of ≥4 points on the NIHSS*.

Concerning post stroke complications, almost all patients in the TS group developed a hospital acquired pneumonia (HAP) (97.5 vs. 37.6%, *p* < 0.001) and rates of failed extubation (37.5 vs. 3.2%, *p* < 0.001), sepsis (27.5 vs. 2.7%, *p* < 0.001), sICH (12.5 vs. 3.9%, *p* = 0.026) as well as decompressive hemicraniectomy (DH) (51.4 vs. 3.8%, *p* < 0.001) were significantly higher.

With exception for diabetes mellitus and symptom-onset-to-recanalization time, all factors described above were predictive for the need of TS in the univariate analysis. As shown in Table 2, HAP [OR 64.6 (8.81–473.41), *p* < 0.001], DH [OR 27.1 (12.44–59), *p* < 0.001], failed extubation [18.16 (8.27–39.9), *p* < 0.001], sepsis [18.16 (8.27–39.9), *p* < 0.001], a history of pulmonary embolism [4.82 (1.83–12.74), *p* = 0.001], no IVT treatment [0.32 (0.14–0.75), *p* = 0.009] and sICH [3.54 (1.26–9.91), *p* = 0.016] were among the strongest predictors for TS.

**Table 2:** Univariate logistic regression of predictive factors for the need of tracheostomy after mechanical thrombectomy.

	**OR (95% CI)**	***p*-value**
Age	0.97 (0.95–0.99)	0.002
Diabetes mellitus	1.94 (0.99–3.82)	0.054
Pulmonary embolism	4.82 (1.83–12.74)	0.001
NIHSS at baseline	1.053 (1–1.11)	0.038
cCT ASPECTS	0.7 (0.57–0.86)	<0.001
cCT ASPECTS 24-h follow-up	0.73 (0.65–0.82)	<0.001
Symptom onset to recanalization time	1 (1–1)	0.070
Intravenous thrombolysis	0.32 (0.14–0.75)	0.009
General anesthesia	3.22 (1.12–9.27)	0.030
Any ICH	3.53 (1.79–6.99)	<0.001
sICH^#^	3.54 (1.26–9.91)	0.016
Subarachnoid hemorrhage	2.98 (1.42–6.26)	0.004
Decompressive hemicraniectomy	27.1 (12.44–59)	<0.001
Sepsis	13.7 (5.84–32.17)	<0.001
Hospital acquired pneumonia^*^	64.6 (8.81–473.41)	<0.001
Failed extubation	18.16 (8.27–39.9)	<0.001

*NIHSS, National institute of health stroke scale; sICH, symptomatic intracerebral hemorrhage*.

# *Symptomatic intracerebral hemorrhage was defined as any intraparenchymal hemorrhage leading to a clinical deterioration of ≥ 4 points on the NIHSS*.

* *Any in-hospital pneumonia being diagnosed at least 48–72 h after admission*.

In multivariate logistic regression, all factors except ASPECTS and age were included. The latter were excluded because of a clear selection bias for age (younger patients are more likely to be selected for TS and older patients are more likely to receive limited therapy) and to avoid multicollinearity [as ASPECTS and NIHSS are highly correlated (*r* = −0.248, *p* < 0.001)]. Using the stepwise backward selection (Wald) function of the logistic regression model, HAP [OR 21.26 (2.76–163.56), *p* = 0.003], DH [OR 9.94 (3.92–25.21), *p* < 0.001], failed extubation [OR 8.41 (3.09–22.93), *p* < 0.001] and sepsis [OR 5.39 (1.71–16.91), *p* = 0.004] remained as strongest predictors for TS (Table 3).

**Table 3:** Multivariate logistic regression model including predictive factors for the need of tracheostomy after mechanical thrombectomy.

	**OR (95% CI)**	***p*-value**
Decompressive hemicraniectomy	9.94 (3.92–25.21)	<0.001
Sepsis	5.39 (1.71–16.91)	0.004
Hospital acquired pneumonia^*^	21.26 (2.76–163.56)	0.003
Failed extubation	8.41 (3.09–22.93)	<0.001

* *Any in-hospital pneumonia being diagnosed at least 48–72 h after admission; stepwise backward selection has been used to exclude predictors with p > 0.1*.

The regression coefficients of the multivariate logistic regression model given in Table 3 were used to create a score for the prediction of TS using the equation given in Supplementary Figure 1. This score, ranging from −7 to 4 points, showed an excellent predictive value for the need for TS (AUROC, 0.929, 95%CI, 0.884–0.974, *p* < 0.001). Patients with TS had a median score of −1 (IQR, −1 to 1), and patients without TS had a median score of −6 (IQR, −6 to −3; *p* < 0.001) points. A cut-off score of −2 points has been identified with a sensitivity of 81% and a specificity of 94%.

As it can be assumed that in a high proportion of LVOS-patients with severe neurological deficits and complications the therapy was limited and therefore no TS has been performed, we conducted a secondary analysis combining patients who died during their neuro-ICU stay (discharge mRS = 6) with TS patients in one group (TS plus severe cases group) and compared these patients to the nTS group. Baseline characteristics are given in Supplementary Table 1, which showed multiple differences, especially concerning clinical scores, imaging characteristics, and complication rates. All these different factors were included in a multivariate logistic regression model with stepwise backward selection, which again revealed HAP, failed extubation, sepsis, and DH as strongest predictors for a combined endpoint TS and death at discharge (Supplementary Table 2).

As shown in Supplementary Table 3, there was no difference in admission pH, paO_2_, paCO_2_, oxygenation index, or oxygen saturation. Patients with TS showed a trend towards higher lactate on admission (1.5 ± 1.2 vs. 1.3 ± 0.7, *p* = 0.062).

In patients with TS, the median time from admission to surgery was 16 days (IQR, 13–19). Patients with TS had a significant longer length of in-hospital stay [22 (14.25–29) vs. 9 (5–14) days, *p* < 0.001] and longer periods of orotracheal tube- [14.22 (12.42–17.36) vs.0.04 (0–0.46) days, *p* < 0.001] and total mechanical ventilation [19.92 (16.38–24.94) vs. 0.04 (0–0.46) days, *p* < 0.001]. The median period from admission to TS was 15.54 (12.95–18.85) days. Longer periods from admission to TS were significantly correlated with longer ICU length of stay (*r* = 0.384, *p* = 0.03), longer periods of orotracheal- (*r* = 0.792, *p* < 0.001) and total period of mechanical ventilation (*r* = 0.445, *p* = 0.004). Patients with TS and favorable clinical outcome (mRS ≤ 3) had no shorter periods from admission to TS [favorable outcome: 17 (IQR, 15–20) days vs. unfavorable outcome: 15 (IQR, 13–9), *p* = 0.425] and there was no significant correlation between the time period (days) from admission to tracheostomy and clinical outcome scores (NIHSS at discharge: *r* = 0.125, *p* = 0.461; mRS at 90 days: *r* = −0.179, *p* = 0.403).

Functional outcome scores were positively correlated with longer periods of orotracheal- (NIHSS at discharge: *r* = 0.478, *p* < 0.001; mRS at 90 days: *r* = 0.256, *p* < 0.001) and total period of mechanical ventilation (see Supplementary Table 4; NIHSS at discharge: *r* = 0.448, *p* < 0.001; mRS at 90 days: *r* = 0.229, *p* < 0.001).

## Discussion

In the present study, we identified HAP, failed extubation, the need for DH, and Sepsis as strong predictors for patients to undergo TS during their neuro-ICU stay after mechanical thrombectomy.

TS is believed to have distinct advantages to ease weaning in patients with severe dysphagia, reduced level of consciousness, and post-stroke complications. These advantages include a lower death space due to a shorter cannula with weaning facilitation, reduced sedatives, and therefore better mobilization and reduced complications like pneumonia and bedsores, and increases patient comfort (12, 13). However, reliable indications if- and when to perform a TS for patients with acute brain injury are not yet established (13). While studies on TS in mixed ICU populations showed conflicting results (14–16) a first prospective, randomized trial by Bösel et al. (9) (SETPOINT study) showed reduced mortality and sedatives associated with early TS in a population with ischemic and hemorrhagic strokes. A follow-up study (SETPOINT 2) in this respect is highly anticipated (8). In contrast to all possible advantages of TS in patients with acute brain injury, the procedural risks of TS (13) in its two forms as percutaneous tracheostomy and surgical tracheostomy in everyday clinical practice must be balanced against its benefits. Therefore, patient selection is key to being able to determine in which patients TS should be performed early and which patients are more likely to benefit from prolonged orotracheal intubation and later extubation trials.

All previously published studies on the role of TS included either mixed ICU populations or mixed patient groups with acute brain injury or subtypes of stroke. Our study aimed to specifically address risk profiles for TS in patients with LVOS after mechanical thrombectomy, who became an important and intensively studied subgroup of stroke patients during the past years. Patients with LVOS with a short symptom to recanalization time, lower NIHSS, higher ASPECTS as well as no hemorrhagic transformation after MT are very likely to be early extubated in contrast to patients with MT/IVT associated early ICHs or large ischemic cerebral areas. Interestingly, we did not observe ischemic stroke-specific parameters or scores as strongest predictors for TS, but factors commonly complicating the clinical course of major ischemic stroke patients like HAP and (possible associated) sepsis. This reflects the finding, that—besides the reduced level of consciousness—in around 35% of cases cardio-respiratory complications lead to an ICU admission of stroke patients (17). In contrast, stroke severity is likely to be reflected by the high predictive value of DH and failed extubation. TS has been reported to be required in up to a third of patients requiring a DH (18) and is usually performed in patients with a reduced level of consciousness with high ICP associated space-occupying cerebral edema and it is highly variable if a patient requiring this surgery can be readily extubated in a short time period after the surgery (19). In addition, failed extubations are highly likely in patients with stroke-induced dysphagia and reduced level of consciousness, being both highly prevalent in LVOS patients (20, 21). Besides dysphagia and reduced level of consciousness also pneumonia contributes to the failure of extubations, prolonged ventilatory support, and TS (4). The risk for pneumonia itself is associated with stroke severity and stroke outcome and has been reported to be as high as 60% in multiple studies (4). In our study, almost all patients in the TS group had pneumonia compared with 37.6% in the nTS group, which is within the reported range of 4% to 56% of all stroke patients being treated in stroke units (4). This high prevalence of pneumonia increases the risk for sepsis again, which in most cases is caused by pneumonia (22).

Taking all thoughts on these contributing factors to indicate a TS into consideration, and overall patient type emerges known to neurointensivists, as all these factors are influencing each other. Patients with severe strokes are at high risk to develop HAP and are likely to require DH, both again leading to failed extubation and to severe systemic inflammatory responses caused by multiple infectious pathways and DH as major surgery itself. These major predicting factors for TS also were directly or indirectly represented in a previously published score to predict the need for TS in a mixed stroke population [Stroke-Related Early Tracheostomy Score (23)]. Our data suggest that especially these kinds of patients are likely to undergo TS and therefore can be scheduled early for this procedure if these risk factors are present. These patients also might represent the subgroup of ischemic stroke patients most likely to benefit from early TS, given the possible benefits mentioned above. In this respect, however, clinical outcome in our study was not correlated with time from admission to TS. This might be explained by the current practice in our hospital that TSs are performed late to give patients the chance to functionally improve and to perform weaning and extubation trials. Therefore, differences in timepoints of TS were small and no possible differences in outcome could have been detected. Another reason could be, that the TS group is too small, and therefore not enough statistical power is present to detect influences of earlier TS on clinical outcome. What we observed, however, was a significant positive correlation between the duration of mechanical ventilation and clinical outcome. Rather of being an epiphenomenon likely to be explained by the fact that patients with complications and more comorbidities are also more likely to require longer ventilatory support, detrimental effects of mechanical ventilation (e.g., barotrauma, immunotrauma triggering systemic inflammatory responses) and prolonged exposure to sedatives itself can contribute to unfavorable outcomes (24, 25). In this respect, as a correlation between mechanical ventilation periods and clinical outcome-, but not between the period from admission to TS and outcome was detected, from our data it can be speculated that not the modality of ventilatory support (TS vs. orotracheal tube) influences functional outcome, but the total time the patient is ventilator dependent. This again supports the notion and current recommendation that weaning should be initiated early and prolonged intubation should be avoided in stroke patients (5).

The strength of our study is the inclusion of a large number of LVOS patients with prospectively derived data, all undergoing MT and being solely treated in a specialized neuro-ICU. However, using this prospectively derived data does not change the retrospective design of this study, which represents one of its major limitations. Other limitations include a single-center study design, only reflecting local practices and procedures associated with the use of TS, the lack of information concerning extend and type of dysphagia as a major contributing factor for the decision to perform TS, and a relatively low number of TS patients in this ischemic stroke subgroup. Another major limitation of this study is represented by a selection bias applicable to age and limitation of medical and surgical therapy according to the presumed and the actual patient will. Therefore, our data must be interpreted with caution and the results only applied in patients wishing full treatment. In addition, the score created with our single-center data must be validated in other datasets and can only be used considering the aforementioned limitations.

In conclusion, the combination of HAP, failed extubation, DH, and sepsis after MT may be useful for patient selection to indicate TS. If, however, earlier TS—and not solely limitation of mechanical ventilation time—translate into better functional outcomes must be determined by prospective trials like the SETPPOINT 2 study.

## Data Availability Statement

The datasets presented in this article are not readily available because data can only be made accessible upon reasonable request. Requests to access the datasets should be directed to ilko.maier@med.uni-goettingen.de.

## Ethics Statement

The studies involving human participants were reviewed and approved by Ethics Commission of the University Medicine Göttingen, Germany. Written informed consent for participation was not required for this study in accordance with the national legislation and the institutional requirements.

## Author Contributions

IM designed the study and was involved in the acquisition and statistical analysis of the data, drafted, finalized the manuscript, and approved the manuscript before submission. KS was involved in the acquisition and statistical analysis of the data and approved the manuscript before submission. MB and JL contributed to the manuscript and approved the manuscript before submission. DB and M-NP contributed to the manuscript, was involved in the acquisition of the data, and approved the manuscript before submission. All authors contributed to the article and approved the submitted version.

## Conflict of Interest

M-NP received speakers' honoraria from Siemens Healthineers. The remaining authors declare that the research was conducted in the absence of any commercial or financial relationships that could be construed as a potential conflict of interest.

## Publisher's Note

All claims expressed in this article are solely those of the authors and do not necessarily represent those of their affiliated organizations, or those of the publisher, the editors and the reviewers. Any product that may be evaluated in this article, or claim that may be made by its manufacturer, is not guaranteed or endorsed by the publisher.
