# Supplementary material for: Predictive Factors for the Need of Tracheostomy in Patients With Large Vessel Occlusion Stroke Being Treated With Mechanical Thrombectomy
Source: Front Neurol. 2021 Nov 26;12:728624. doi: 10.3389/fneur.2021.728624 (PMC8660673; doi:10.3389/fneur.2021.728624)
Supplement: Supplementary file 1 [file Table_1.DOCX]

| Supplementary table 1: Baseline characteristics of a combined group of severe cases (patients with tracheostomy and deceased patients during neuro-ICU therapy) and of patients without tracheostomy after mechanical thrombectomy (n=635) | | | | |
| --- | --- | --- | --- | --- |
|  |  |  |  |  |
|  |  | Severe cases (patients with tracheostomy combined with deceased patients) (n=155) | No tracheostomy group (n=440) | p-value |
| Patient characteristics and past medical history | |  |  |  |
|  | Age (median, IQR) | 77 (70-84) | 76 (64-82) | 0.022 |
|  | Sex (n, % male) | 64 (41.3) | 223 (50.7) | 0.279 |
|  | Arterial hypertension (n, %) | 127 (81.9) | 364 (82.7) | 0.224 |
|  | Hyperlipoproteinemia (n, %) | 61 (39.4) | 237 (53.9) | 0.040 |
|  | Diabetes mellitus (n, %) | 50 (32.3) | 127 (28.9) | 0.137 |
|  | Atrial fibrillation (n, %) | 76 (49) | 202 (45.9) | 0.135 |
|  | Peripheral artery disease (n, %) | 11 (7.1) | 26 (5.9) | 0.422 |
|  | Obesity (n, %) | 32 (20.6) | 132 (30) | 0.135 |
|  | Smoking (n, %) | 16 (10.3) | 85 (19.3) | 0.036 |
|  | Coronary artery disease (n, %) | 41 (26.5) | 102 (23.2) | 0.171 |
|  | Chronic renal failure (n, %) | 44 (28.4) | 99 (22.5) | 0.024 |
|  | Congestive heart failure (n, %) | 42 (27.1) | 118 (26.8) | 0.414 |
| Pulmonary disease* (n, %) | | 43 (27.7) | 84 (19.1) | 0.005 |
|  | COPD (n, %) | 26 (16.8) | 34 (7.7) | <0.001 |
|  | Bronchial asthma (n, %) | 3 (1.9) | 9 (2) | 0.951 |
|  | Lung cancer (n, %) | 2 (1.3) | 12 (2.7) | 0.379 |
|  | Pulmonary emphysema (n, %) | 1 (0.6) | 7 (1.6) | 0.435 |
|  | Pulmonary embolism (n, %) | 15 (9.7) | 12 (2.7) | <0.001 |
|  | Community acquired pneumonia (n, %) | 2 (1.3) | 6 (1.4) | 0.962 |
|  | Pulmonary fibrosis (n, %) | 2 (1.3) | 1 (0.2) | 0.086 |
|  | Pulmonary hypertension (n, %) | 6 (3.9) | 11 (2.5) | 0.282 |
| Clinical scores and imaging characteristics | |  |  |  |
|  | NIHSS baseline (median, IQR) | 17 (13-21) | 14 (9-18) | <0.001 |
|  | NIHSS discharge (median, IQR) | 19 (13-22.25) | 5 (2-11) | <0.001 |
|  | cCT ASPECTS at baseline (median, IQR) | 8 (7-9) | 7 (6-9) | <0.001 |
|  | cCT ASPECTS 24-hour follow-up (Median, IQR) | 3 (1-6) | 7 (6-8) | <0.001 |
|  | Symptom onset to recanalization time (median min, IQR) | 242 (189.75-312.5) | 203.5 (149-279) | <0.001 |
|  | Successful recanalization (n, %) | 103 (66.5) | 385 (87.5) | <0.001 |
|  | Final mTICI score (median, IQR) |  |  | 0.001 |
|  | mTICI 0 | 22 (14.2) | 30 (6.8) |  |
|  | mTICI 1 | 10 (6.5) | 15 (3.4) |  |
|  | mTICI 2a | 19 (12.3) | 33 (7.5) |  |
|  | mTICI 2b | 49 (31.6) | 165 (37.5) |  |
|  | mTICI 2c | 27 (17.4) | 96 (21.8) |  |
|  | mTICI 3 | 27 (17.4) | 123 (28) |  |
|  | Oxygenation index (Median, IQR) | 343.5 (239.21-503.75) | 380 (300-502) | 0.034 |
| Ischemic stroke treatment characteristics | |  |  |  |
|  | Side of occluded vessel (n, % right) | 55 (35.5) | 206 (46.8) | 0.464 |
|  | Site of occluded vessel |  |  | <0.001 |
|  | M1 (n, %) | 63 (40.6) | 237 (53.9) |  |
|  | M2 (n, %) | 12 (7.7) | 73 (16.6) |  |
|  | ICA proximal (n, %) | 7 (4.5) | 14 (3.2) |  |
|  | Intracranial carotid bifurcation (n, %) | 49 (31.6) | 83 (18.9) |  |
|  | BA (n, %) | 22 (14.2) | 38 (8.6) |  |
|  | Other (n, %) | 2 (1.3) | 21 (4.8) |  |
| Stroke etiology (TOAST criteria) | |  |  | 0.129 |
|  | Large vessel atherosclerosis | 9 (5.8) | 58 (13.2) |  |
|  | Cardioembolism | 73 (47.1) | 221 (50.2) |  |
|  | Stroke of other determined etiology | 4 (2.6) | 18 (4.1) |  |
|  | Stroke of undetermined etiology | 56 (36.1) | 151 (34.3) |  |
|  | Missing (n, %) |  |  |  |
|  | Wake up stroke (n, %) | 19 (12.3) | 49 (11.1) | 0.416 |
|  | Intravenous thrombolysis (n, %) | 52 (33.5) | 180 (40.9) | 0.030 |
|  | Type of anesthesia for mechanical thrombectomy |  |  | 0.001 |
|  | General anesthesia (n, %) | 106 (68.4) | 201 (45.7) |  |
|  | Conscious sedation (n, %) | 22 (14.2) | 108 (24.5) |  |
|  | Switch from conscious sedation to general anesthesia (n, %) | 12 (7.7) | 38 (8.6) |  |
| Postinterventional complications | |  |  |  |
|  | Failed extubation (n, %) | 25 (16.1) | 9 (2) | <0.001 |
|  | Hospital acquired pneumonia (n, %) | 88 (56.8) | 175 (39.8) | <0.001 |
|  | Sepsis (n, %) | 22 (14.2) | 5 (1.1) | <0.001 |
|  | Any ICH (n, %) | 47 (30.3) | 51 (11.6) | <0.001 |
|  | Symptomatic ICH^#^ (n, %) | 20 (12.9) | 7 (1.6) | <0.001 |
|  | Subarachnoid hemorrhage (n, %) | 38 (24.5) | 37 (8.4) | <0.001 |
|  | Decompressive hemicraniectomy (n, %) | 29 (18.7) | 13 (3) | <0.001 |
| COPD: Chronic obstructive pulmonary disease; NIHSS: National institute of health stroke scale; ASPECTS: Alberta stroke programme early CT score; mTICI: modified Thrombolysis in cerebral infarction scale; M1/2: medial cerebral artery in its M1 or M2 segment; ICA: internal carotid artery, BA: basilar artery, TOAST: Trial of Org 10172 in Acute Stroke Treatment; ICH: Intracerebral hemorrhage *Pulmonary disease includes COPD, bronchial asthma, lung cancer, pulmonary emphysema, pulmonary fibrosis, pulmonary hypertension and pulmonary embolism; Successful recanalization was defined as mTICI ≥ 2b; ^#^ Symptomatic intracerebral hemorrhage was defined as any intraparenchymal hemorrhage leading to an clinical deterioration of ≥ 4 points on the NIHSS | | | | |
