# Supplementary material for: Predictive Factors for the Need of Tracheostomy in Patients With Large Vessel Occlusion Stroke Being Treated With Mechanical Thrombectomy
Source: Front Neurol. 2021 Nov 26;12:728624. doi: 10.3389/fneur.2021.728624 (PMC8660673; doi:10.3389/fneur.2021.728624)
Supplement: Supplementary file 2 [file Table_2.DOCX]

| Supplementary table 2: Multivariate logistic regression model including predictive factors for the combined endpoint need of tracheostomy and death during neuro-ICU stay | | |
| --- | --- | --- |
|  |  |  |
|  | OR (95% CI) | p-value |
| Decompressive hemicraniectomy | 7.70 (2.54-23.30) | <0.001 |
| Sepsis | 4.25 (1.13-16.08) | 0.033 |
| Hospital acquired pneumonia* | 13.34 (1.65-107.83) | 0.015 |
| Failed extubation | 8.86 (2.65-29.58) | <0.001 |
| ICU: intensive care unit, OR: odds ratio, CI: confidence interval. *Any in-hospital pneumonia being diagnosed at least 48–72 hours after admission | | |
|  |  |  |
