# Supplementary material for: Predictive Factors for the Need of Tracheostomy in Patients With Large Vessel Occlusion Stroke Being Treated With Mechanical Thrombectomy
Source: Front Neurol. 2021 Nov 26;12:728624. doi: 10.3389/fneur.2021.728624 (PMC8660673; doi:10.3389/fneur.2021.728624)
Supplement: Supplementary file 3 [file Table_3.DOCX]

| Supplementary table 3: Results of blood gas analysis and oxygenation on admission | | | |
| --- | --- | --- | --- |
|  |  |  |  |
|  | Tracheostomy group (n=40) | No tracheostomy group (n=595) | p-value |
| pH (± SD) | 7.38 ± 0.08 | 7.38 ± 0.35 | 0.961 |
| paCO2 (± SD) | 41.6 ± 7.8 | 40.8 ± 8 | 0.553 |
| paO2 (± SD) | 140.3 ± 87.2 | 127.3 ± 71.4 | 0.277 |
| paO2/paCO2 (± SD) | 383 ± 278 | 427 ± 216 | 0.242 |
| Lactate (± SD) | 1.5 ± 1.2 | 1.3 ± 0.7 | 0.062 |
| spO_2_ (%) | 98 | 97 | 0.331 |
|  |  |  |  |
