# Supplementary material for: Predictive Factors for the Need of Tracheostomy in Patients With Large Vessel Occlusion Stroke Being Treated With Mechanical Thrombectomy
Source: Front Neurol. 2021 Nov 26;12:728624. doi: 10.3389/fneur.2021.728624 (PMC8660673; doi:10.3389/fneur.2021.728624)
Supplement: Supplementary file 4 [file Table_4.DOCX]

| Supplementary table 4: Correlations between treatment periods and functional outcome of patients with- and without tracheostomy after mechanical thrombectomy | | | | | | | | |
| --- | --- | --- | --- | --- | --- | --- | --- | --- |
|  |  |  |  |  |  |  |  |  |
|  | NIHSS at discharge | |  | mRS at discharge | |  | mRS 90 days | |
|  | Correlation coefficient | p-value |  | Correlation coefficient | p-value |  | Correlation coefficient | p-value |
| Time from admission to tracheostomy (n=40) | 0.125 | 0.461 |  | -0.045 | 0.788 |  | -0.179 | 0.403 |
| In-hospital length of stay (n=635) | 0.312 | <0.001 |  | 0.098 | 0.019 |  | -0.020 | 0.676 |
| Length of mechanical ventilation (tube, n=635) | 0.478 | <0.001 |  | 0.385 | <0.001 |  | 0.256 | <0.001 |
| Length of mechanical ventilation (total, n=635) | 0.448 | <0.001 |  | 0.339 | <0.001 |  | 0.229 | <0.001 |
| NIHSS: National Institute of Health Stroke Scale: mRS: modified Rankin scale | | | | | | | | |
|  |  |  |  |  |  |  |  |  |
