# Supplementary material for: Predictive Factors for the Need of Tracheostomy in Patients With Large Vessel Occlusion Stroke Being Treated With Mechanical Thrombectomy
Source: Front Neurol. 2021 Nov 26;12:728624. doi: 10.3389/fneur.2021.728624 (PMC8660673; doi:10.3389/fneur.2021.728624)
Supplement: Supplementary file 5 [file Data_Sheet_1.docx]

$$Tracheostomy score= -6.088+\left( 2.322*DH \right)+\left( 1.635*Sepsis \right)+\left( 3.073*HAP \right)+(2.148*failed extubation)$$

**Supplementary figure 1:** Equation to create a tracheostomy score using the regression coefficients of a multivariate logistic regression model including factors with the highest predictive value for tracheostomy after mechanical thrombectomy. For the predictors either zero (predictor not present) or one (predictor present) must be inserted. A cut of score of -2 points has been identified with a sensitivity of 81% and a specificity 94%. DH: decompressive hemicraniectomy, HAP: hospital acquired pneumonia.
